# Study on the involvement of soluble guanylyl cyclase and its different isoforms in carbon monoxide and carbon monoxide releasing molecule-2 induced vasodilatation

**DOI:** 10.1186/1471-2210-11-S1-P20

**Published:** 2011-08-01

**Authors:** Kelly Decaluwé, Bart Pauwels, Sara Verpoest, Robrecht Thoonen, Emmanuel Buys, Peter Brouckaert, Johan Van de Voorde

**Affiliations:** 1 Department of Pharmacology, Ghent University, Ghent, Belgium; 2 Department for Molecular Biomedical Research, VIB, Ghent, Belgium; 3 Department of Biomedical Biology, Ghent University, Ghent, Belgium; 4 Anesthesia Center for Critical Care Research, Department of Anesthesia and Critical Care, Massachusetts General Hospital, Harvard Medical School, Boston, MA, USA

## Background

Besides nitric oxide, carbon monoxide (CO) also activates soluble guanylyl cyclase (sGC). CO as well as the CO-donor CORM-2 have been shown to possess vasodilatory properties. Whether these vasodilatory properties by CO can be attributed to sGC activation is still a matter of debate. The aim of this study was to examine the involvement of sGC and its different subunits in CO and CORM-2 induced vasodilatation within different vascular tissues.

## Materials and methods

Isometric tension recordings were performed using mice isolated aortic rings, femoral artery ring segments as well as corpora cavernosa (CC). To be able to distinguish between the different sGC subunits we evaluated responses to saturated CO solutions and CORM-2 in both sGCa_1_^-/-^ and sGCβ_1_^KI/KI^ mice and their wild-type controls.

## Results

Saturated CO solution was unable to relax mice isolated blood vessels, whereas it induced concentration-dependent relaxations in mice CC. In CC of wild-type mice, the response to CO was completely inhibited by the sGC inhibitor ODQ. The involvement of sGC in the CO-induced corporal relaxation was further confirmed by the loss of response to CO in CC isolated from sGCβ_1_^KI/KI^ mice. Moreover, the vasodilatory responses of CO in the corporal tissue of sGCa_1_^-/-^ mice were strongly inhibited although not completely abolished. In contrast to CO, CORM-2 was able to relax all vascular tissues examined in the present study, although ODQ only partially blocked the response to CORM-2 in the aorta. Interestingly ODQ did not affect the CORM-2 induced relaxation in the femoral arteries and the CC, indicating that sGC is not involved, which was confirmed using the transgenic mice.

## Conclusion

This study clearly illustrates that the molecular mechanism of CORM-2 induced vasorelaxation differs from that of CO induced vasorelaxation. While the CO induced vasorelaxation depends on activation of sGC, primarily the sGCa_1_β_1_ heterodimer, the vasorelaxing properties of CORM-2 are only partially dependent or even completely independent upon sGC activation. The observation that CO is more effective in relaxing CC tissues than other cardiovascular tissues investigated in the present study suggests that the heme-oxygenase/CO pathway may present a potential new target for therapeutic approaches towards erectile dysfunction.

